# Synthesis of Bis(cyclic carbonates) from Epoxy Resin under Microwave Irradiation: The Structural Analysis and Evaluation of Thermal Properties

**DOI:** 10.3390/molecules29010250

**Published:** 2024-01-03

**Authors:** Edyta Hebda, Jan Ozimek, Kinga Szołdrowska, Krzysztof Pielichowski

**Affiliations:** Department of Chemistry and Technology of Polymers, Cracow University of Technology, Warszawska 24, 31-155 Krakow, Poland; jan.ozimek@pk.edu.pl (J.O.); kinga.szoldrowska@student.pk.edu.pl (K.S.)

**Keywords:** cyclic carbonate, carbon dioxide, epoxy resin, CO_2_ utilization, thermal properties

## Abstract

This article describes the use of microwave irradiation in the synthesis of bis(cyclo carbonate) compounds (BCCs) in bulk (without solvent) from carbon dioxide capture using an epoxidized compound—a commercial epoxy resin—and compares this process to the conventional method. CO_2_ cycloaddition to epoxides is an ecological and efficient method for the formation of bis(cyclic carbonates). Moreover, the introduction of gas into the reaction mixture was carried out at atmospheric pressure with a controlled flow rate, which is advantageous from an economic point of view. Progressive structural changes and the presence of characteristic chemical groups were monitored using attenuated total reflectance infrared spectroscopy with Fourier transform. The obtained crude products were purified to obtain three fractions, which were subjected to detailed structural analysis using FT-IR and ^13^CNMR. The formation of the main product with two cyclic carbonates was confirmed. The presence of monomers, dimers and trimers in individual fractions as well as their thermal stability were determined, and the molecular masses in individual fractions were determined using gel permeation chromatography (GPC).

## 1. Introduction

Carbon dioxide (CO_2_) is one of the gaseous components of the atmosphere occurring naturally, but it is also emitted as a result of the action of anthropogenic factors, and it belongs to the group the so-called greenhouse gases (greenhouse gases, GHGs) [1]. The continuous increase in CO_2_ concentration in the atmosphere is correlated with the industrial revolution, the conventional beginning of which dates back to 1750. At that time, the concentration of this gas was approximately 277 ppm, while today this value is above 400 ppm [2,3]. The increase in CO_2_ concentration is mainly caused by energy production methods based on the combustion of fossil fuels. The high concentration of CO_2_ in the atmosphere has led to a number of environmental problems, in particular the increasing greenhouse effect and global warming of the Earth’s climate. The report of the Intergovernmental Panel on Climate Change (IPCC) indicates that the concentration of CO_2_ in the atmosphere by 2100 may be as high as 570 ppm, which will also be associated with an increase in the average global temperature by approximately 1.5–2.0 °C and an increase in the sea level by approximately 0.38 m [4]. Due to an increased emission of the greenhouse gases, research and development work in the field of capturing, storing and using greenhouse gases, in particular in the synthesis of organic compounds, has become justified from an environmental and economic point of view. The developing market of chemical raw materials based on carbon dioxide is primarily caused by the commercialization of ecological, non-phosgene synthesis methods, as well as the possibility of obtaining high-quality products necessary for potential applications. The disadvantage of phosgene-based processes, which occur at high speed due to the high reactivity of this compound, are limitations related to work safety and the production of large amounts of harmful by-products [5]. One of the most important applications of CO_2_ is the synthesis of five-membered cyclocarbonates through the catalytic conversion of epoxy compounds [6,7]. These cyclocarbonates have a wide range of applications, such as aprotic polar solvents, fuel additives, electrolytes and intermediates for polymer synthesis [8,9].

Recently, bis-cyclocarbonates have been used on a large scale to obtain non-isocyanate polyurethanes (NIPUs), which are an alternative to conventional polyurethanes synthesized using toxic isocyanates. The main advantages of NIPUs are better chemical, mechanical and thermal resistance, and additionally, their synthesis process is insensitive to moisture, which is a significant technological advantage compared to conventional PU synthesis [10]. With the exception of some mechanical properties comparable to conventional PU, NIPUs typically exhibit increased chemical resistance and lower permeability, as well as improved water absorption and thermal stability [11]. Moreover, NIPUs are insensitive to moisture in the surrounding environment [12]. These properties give NIPUs many potential applications, such as chemical resistant coatings, paints and sealants [13,14,15,16,17,18,19,20,21,22].

At the end of the 1980s, methods of conducting chemical reactions in the microwave radiation field aroused particular interest [23]. One of the advantages of microwave-supported processes is a significant reduction in the reaction time compared to conventional methods. Currently, there are two theories that describe the influence of microwave radiation on the course of chemical reactions. According to one of them, although the course of chemical reactions is many times faster than under conventional heating conditions, the kinetics and mechanism of the chemical reaction remain unchanged. It is assumed that under microwave conditions there is a sudden, uncontrolled increase in temperature and this is the reason for the increase in the reaction rate. The second theory assumes the occurrence of a specific microwave activation effect causing an increase in the reaction rate that is inadequate for the process temperature. This effect is commonly called the non-thermal microwave effect or specific microwave effect [24,25,26]. There have been several studies in the literature on the use of microwave radiation for the synthesis of NIPUs, mainly to shorten the reaction time. Hwang et al. [25] determined that the reaction of opening carbonate with diamine in a polar solvent, i.e., ethyl lactation under microwave radiation, leads to obtaining a NIPU oligomer with a high molecular weight of approx. 15,000 g/mol within 1 h. Quérette et al. [18] investigated the production of polyhydroxyurethane nanoparticles by nanoprecipitation. First, they obtained PHU catalytically by reacting sebacic bis(cyclic carbonate) with aliphatic diamines (hexamethylenediamine and tetramethylenediamine) under microwave radiation, which contributed to shortening the reaction time. The obtained PHU was characterized by the presence of hydroxyl groups, mainly secondary ones, and a molecular weight of 7000 g/mol; finally, PHU nanoparticles were obtained by nanoprecipitation, using DMSO as a solvent. Research on increasing the scale of NIPU production under microwave radiation was conducted by Yang et al. [27]. They demonstrated that the use of a continuously operating tubular reactor ensures a low TOE (Turn Over Energy) level while obtaining homogeneous NIPU materials without batch differences.

Bis-cyclocarbonates used for the synthesis of NIPUs can be obtained by the reaction of epoxy resin with carbon dioxide. The method is easy to perform and allows for achieving a high degree of conversion of the main product; however, in the case of compounds with higher molecular weights, such as epoxy resins, it is time-consuming [28]. Syntheses of monocarbonates obtained under microwave radiation have already been described in the literature [29,30,31]; however, to the best of our knowledge, there are no studies on the synthesis of bis-cyclocarbonates with higher molecular weights from epoxy resins.

Therefore, the aim of this work was to develop the solvent-free synthesis of bis-cyclocarbonates from epoxy resins using CO_2_ under microwave radiation. The next task was to isolate and to purify the obtained bis-cyclocarbonate in order to obtain a homogeneous product, which may finally contribute to improving the properties of NIPUs.

## 2. Results and Discussion

**Figure 1:**
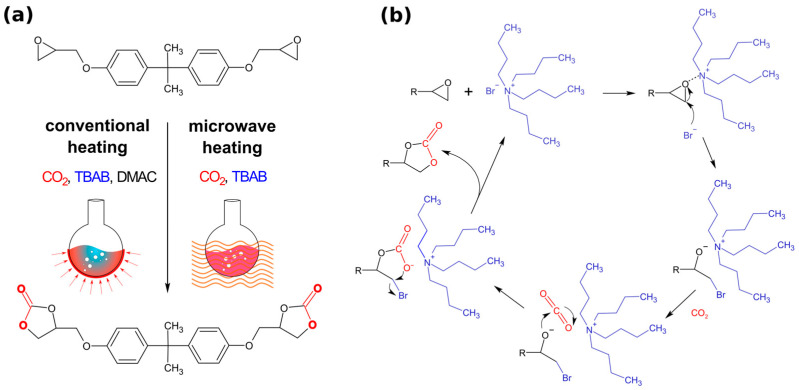
(**a**) Synthesis of BCCBPA from epoxy resin BPA carried out under conventional conditions and with the use of microwave radiation. (**b**) Proposed mechanism of the catalytic fixation of carbon dioxide with epoxides (adopted from references [33,34]).

We have investigated the influence of the type of heating, conventional or microwave, on the rate of obtaining bis(cyclic carbonate) as a result of the cycloaddition of CO_2_ with epoxides. The catalytic binding of CO_2_ to epoxides, catalyzed by a quaternary ammonium salt (TBAB), was carried out at a constant gas flow at a temperature of 105 °C using the commercial epoxy resin Epidian 6 (BPA). It should be emphasized that it was a solvent-free reaction in the case of the reaction carried out using microwaves, and at the end of the reaction performed under conventional heating, 10 mL of solvent was added due to the significant increase in the viscosity of the reaction mixture. In both cases, the reaction was carried out at atmospheric pressure. Conducting the reaction under microwave radiation was aimed at maximally increasing the temperature of the reaction mixture, the heat of which spreads evenly throughout its entire volume in a very short time with high energy efficiency [25,26,32]. Figure 1 shows the synthesis route of bis(cyclocarbonate) Bisphenol A (BCCBPA) from BPA resin (Epidian 6), including the suggested mechanism of the catalytic fixation of CO_2_ with epoxides.

The mechanism of this reaction in the presence of quaternary ammonium salts has already been described in the literature [33,34]. Catalysts play an important role in this process and should have good nucleophilicity. Initially, the reaction involves a nucleophilic attack of the anion on the epoxy ring. The resulting alkoxide reacts with CO_2_. The resulting open-chain carbonate undergoes intramolecular displacement according to the SN2 nucleophilic substitution reaction mechanism. As a result of the ring closing reaction, cyclic carbonate groups are formed [35].

**Figure 2:**
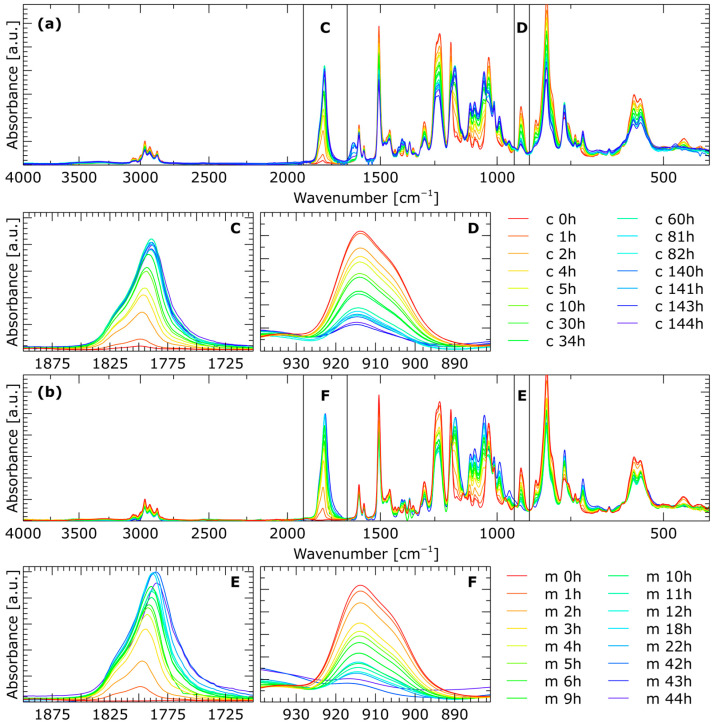
FT-IR spectra taken to monitor the progress of reaction carried out (**a**) under conventional conditions and (**b**) using microwave irradiation. Graphs **C** and **D** are approximations of the bands at 1800 cm^−1^ and 915 cm^−1^, respectively, for the reaction carried out conventionally, while plots **E** and **F** are approximations of the bands at 1800 cm^−1^ and 915 cm^−1^, respectively, for the reaction carried out in microwave conditions.

The reaction between CO_2_ and epoxides leads to a five-membered ring containing cyclic carbonates. The progress of the reaction was monitored by FT-IR spectroscopy. This technique allowed for regular testing of the samples at various intervals. Figure 2 shows FT-IR spectra of the products obtained both in conventional reactions and under microwave irradiation conditions. A new, gradually growing band appears in all spectra, at approximately 1800 cm^−1^, originating from the stretching vibrations of the C=O carbonyl group of the forming carbonates. At the same time, with the increase in carbonate groups, the band at approximately 915 cm^−1^, originating from oxirane groups present in the resin, decreases as a result of binding with CO_2_. This band is also attributed to bending vibrations coming from CH_3_ groups [36].

**Figure 3:**
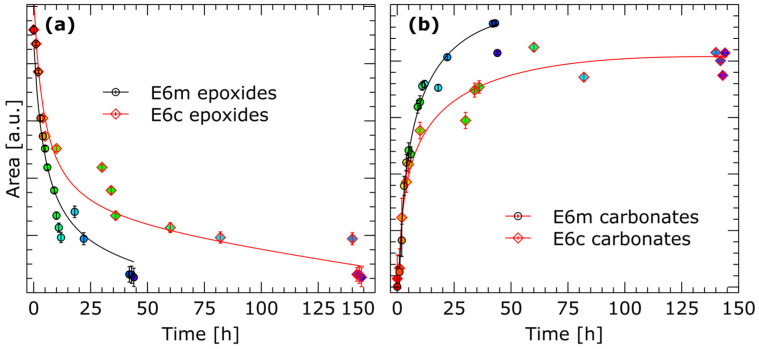
(**a**) Disappearance of absorption intensity for epoxy groups. (**b**) Increase in intensity of formed cyclic carbonates under conventional and microwave conditions (e6c and e6m, respectively) as a function of time in FT-IR. The color of each point correspond to color of the spectra presented in Figure 2.

**Figure 4:**
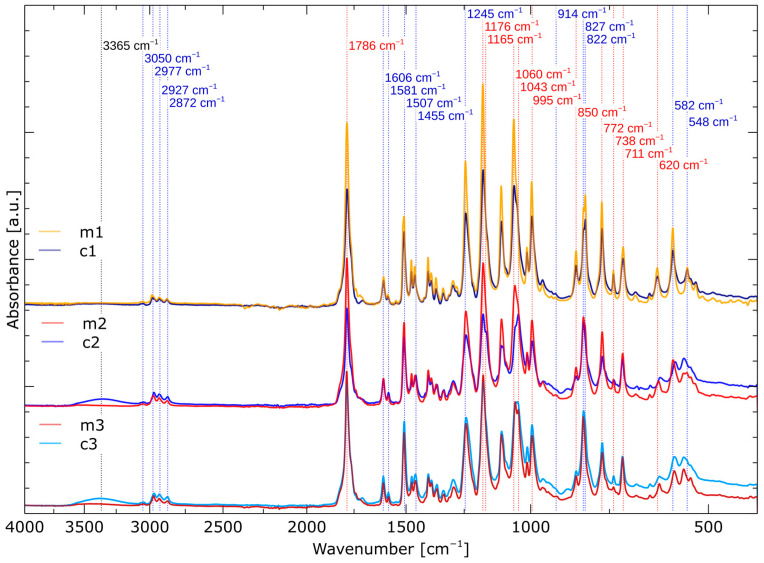
IR spectra of obtained products from each fraction. Blue wavelengths—original bands for Epidian 6, red—bands altered or emerged during the reaction and black—impurities.

In Figure 3, we can see that both the reaction of epoxy groups and the formation of cyclic carbonate groups occur faster when the reaction is carried out under microwave irradiation, compared to the reaction carried out conventionally. This can be explained by better heat distribution throughout the reaction mixture and, consequently, shorter reaction time, i.e., 44 h. In the case of conventional heating, a similar reaction was achieved only after 144 h. The obtained crude BCCBPA was purified to obtain three fractions (m1, m2 and m3 obtained in the microwave condition and c1, c2 and c3 obtained in the conventional condition) differing in weight and purity. Some differences can be observed between each faction, which are visible on the FT-IR spectra—Figure 4.

Some differences can be observed between each faction. One can clearly see OH vibrations extending at the level of 3050 cm^−1^ from the opening of the cyclic carbonate ring or moisture. The fact that it is visible in conventionally prepared c2 and c3 suggests that moisture is more difficult to remove if DMAC is used in the reaction. We did not observe significant changes in the νCH area either for aromatics (3050 cm^−1^) or aliphatic ones (2977 cm^−1^ νCH_3_ asym, 2927 cm^−1^ νCH_2_ asym and 2872 cm^−1^ νCH_3_ asym). Moreover, differences in the intensity of νC=O (1786 cm^−1^) from cyclic carbonates are visible. Generally, the bands originating from cyclic carbonates are more intense in the m1–3 products; however, m3 and c3 are close to each other. Later, we observed four bands from νC=C in aromatics (1606, 1581, 1507 and 1455 cm^−1^). The C-O stretching region is also similar in terms of intensity changes such as νC=O. First, the 1245 cm^−1^ band from νC-O in the aromatic neighborhood is visible, then two pairs of overlapping bands, 1176 with 1165 cm^−1^ and 1060 with 1043 cm^−1^, occur. These pairs originate from asymmetric and symmetric νC-O vibrations and refer to mono- and oligomers, respectively. It can be seen that the CC monomer bands dominate over the oligo-derived bands, especially in m1, m2 and c1, while in c2 and m3 they are at similar heights. The amount of oligomers in the product appears to increase from fraction to fraction, especially in those obtained conventionally. We then observed the stretching band directly in the CC ring (995 cm^−1^). A band located at a distance of 914 cm^−1^ from the bend of the unreacted epoxy ring is barely visible, which suggests that the amount of residual epoxy resin is small in all fractions. We can also see a bending band in the benzene ring at 850 cm^−1^ and two bands resulting from asymmetric out-of-plane bending of the *p*-substituted benzene ring in the monomer (827 cm^−1^) and oligomers (822 cm^−1^). Then, three skeletal vibration bands of the CC ring appear (772, 738 and 711 cm^−1^). Finally, the spectrum is completed by in-plane C=O bending and symmetric out-of-plane vibration of the *p*-substituted benzene ring. Here, from fraction to fraction, we can also observe a broadening of these peaks, suggesting an increased amount of oligomeric CC.

The amount of cyclic carbonates (Table 1) was estimated from FT-IR spectra according to the method described in Section 3.5.

The obtained values show a similar trend, i.e., for both the conventional and microwave methods, the amount of estimated cyclocarbonates decreases and then increases slightly in m3 and c3. However, in m1–m2, the CC content is higher than in fractions c1–c3. This may be due to the higher purity of the obtained bis(cyclocarbonates).

**Figure 5:**
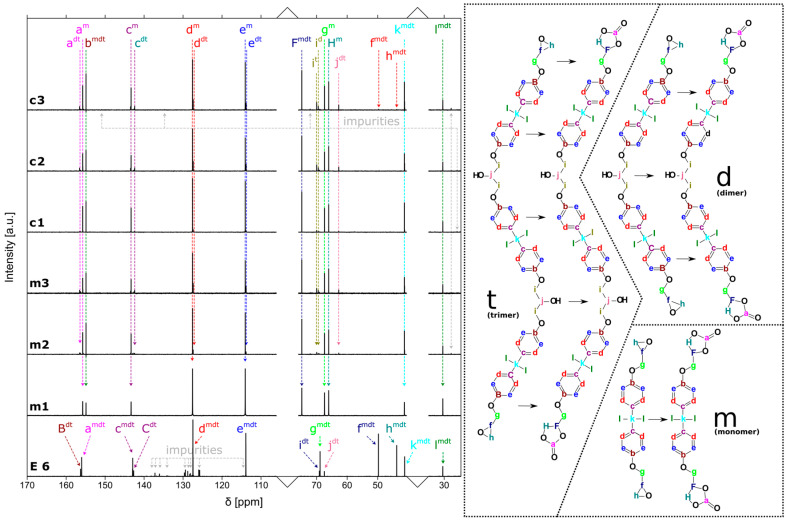
^13^C NMR spectra of bis(cyclic carbonates) after purification in three fractions. The letter is assigned to a specific carbon atom in the molecule, the superscript is monomer, dimer or trimer.

The results of the ^13^C NMR analysis of Epidian 6 and the separated fractions resulting from the purification of the obtained bis(cyclocarbonate) are presented in Figure 5. Among the characteristic signals in the spectrum of Epidian 6, there are peaks (49.76 and 43.78 ppm) coming from carbons associated with oxirane groups. There are also signals at 68.87 ppm (from the C-O bond); 156.02, 142.96, 127.47 and 113.89 ppm (from atoms located in aromatic rings); and signals at 41.19 and 30.71 ppm (from CH_3_ groups). In addition to the monomer, the spectrum of Epidian 6 shows numerous signals also indicating the presence of dimers and trimers. As a result of the reaction, the epoxy groups disappear and cyclic carbonate groups are formed (155.75, 74.84 and 66.05 ppm), which indicates that the reaction has occurred. Comparing the fractions separated from the crude product of the reaction carried out under microwave and under conventional radiation conditions, it can be seen that fraction I is the purest and consists only of monomers. This is evidenced by the lack of a signal at 63.28 ppm assigned to the C-O bond in the C-OH moiety connecting the monomers together. Fraction II also contains small amounts of dimers and trimers, while fraction III has the largest number of them. Only in the case of BCCBPA obtained under conventional conditions are small amounts of unreacted resin visible. Additionally, other impurities are present in this fraction. The purest fraction is m1, while in k1 there are already bands assigned to the C-OH bond occurring in dimers and trimers (at 70 and 71 ppm, respectively). For carbonates obtained under microwave conditions, these bands appear only in the second fraction, i.e., in m2.

**Figure 6:**
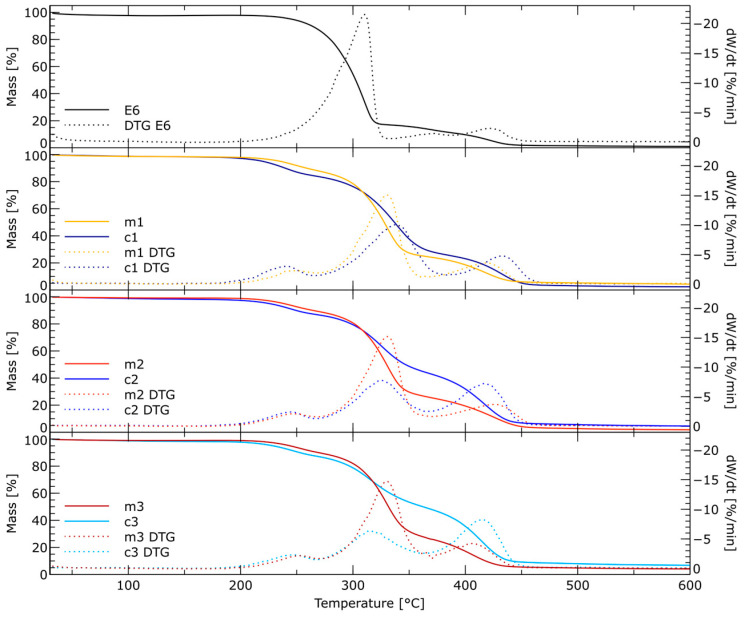
TGA and derivative thermogravimetry (DTG) curves of the synthesized bis(cyclic carbonates) obtained in the conventional and microwave conditions after purification in three fractions.

Thermogravimetric analysis (TGA) was carried out to determine the materials’ thermal stability. Thermogravimetric analyses were carried out to characterize the thermal stability of the epoxy resin and the synthesized bis(cyclic carbonates) obtained after purification in three batches. Figure 6 shows the TG and DTG curves for samples analyzed in a nitrogen atmosphere, while the numerical data are summarized in Table 2. The degradation of E6 resin begins at a temperature of 276.5 °C. In the first stage, the ether bonds between the oxirane moiety and bisphenol A disintegrate, while in the second stage of thermal decomposition, starting from a temperature of about 396.6 °C, the fragments originating from Epidiane decompose. The solid E6 residue at 600 °C is 1%. All bis(cyclic carbonates) obtained in three batches showed thermal stability up to approximately 200 °C. A three-stage decomposition of carbonates is observed in each batch, obtained by conventional and microwave methods. The first stage of thermal decomposition involves the pyrolysis of carbonates, and then there may also be a loss of mass attributed to C=O evolution, or secondary decomposition products, e.g., acetaldehyde [37]. It occurs for samples m1, m2 and m3 at temperatures 222.5, 212.1 and 222.09 °C, respectively, while for c1, c2 and c3, these values are lower and amount to 211.7, 212.1 and 215.4 °C, respectively. It follows that bis(cyclic carbonates) obtained using microwave radiation are characterized by higher thermal stability. During the second stage of degradation, in which the ether bonds break down, the maximum temperature is approximately 330 °C for m1, m2 and m3, while these temperatures are more diverse for c1, c2 and c3 and are 338.1, 324.9 and 318.1 °C, respectively.

Comparing the thermal stability of products obtained by conventional and microwave methods, large differences in mass losses are visible. The maximum mass losses for m1, m2 and m3 are at a similar level (−63.8, −64.2 and −63.8%, respectively), while for k1, k2 and k3, much smaller mass losses of −58,4, −43.0 and −38.0% are observed, respectively. The blurring of this peak suggests lower homogeneity of the tested samples k1, k2 and k3 resulting from the presence of not only mono- but also di- and trimers, which is also confirmed by NMR spectra. The third stage of degradation is the breakdown of fragments coming from Epidiane. In this stage, the maximum degradation temperature and mass loss change, depending on the synthesis method. For samples obtained under microwave radiation, the average maximum temperature and the average mass loss between fractions do not differ significantly and are 413.9 °C and −22.5%, respectively. In the case of bis(cyclic carbonates) obtained using the conventional method, the average maximum temperature (424.5 °C) and the average weight loss (−35.2%) are higher than the same parameters for samples synthesized in a microwave. Additionally, significant differences are visible between individual fractions, which for c1, c2 and c3 are 426.2, 432.4 and 415.0 °C, respectively. These differences may be due to the higher content of dimers and trimers in individual fractions, in addition to monomers.

Finally, GPC analysis was performed for samples m1–m2 (Table 3), because in the previously described studies, they showed greater purity compared to samples c1–c2. 

In each tested sample, there are three peaks corresponding to the masses: monomer, dimer and trimer (504, 731 and 1052 Da, respectively). The highest monomer content (93.6%) was found in sample m1, which coincides with ^13^C NMR and TG analysis. However, the largest share of dimers (15.4%) and trimers (6.1%) was found in sample m2. The analysis confirmed that it is possible to purify crude BCCBPA to separate the monomer.

## 3. Materials and Methods

### 3.1. Materials

Commercially available Bisphenol A (BPA) epoxy resin Epidian 6 (Ciech, Sarzyna, Poland) with a molecular weight of 360 g mol^−1^ was used. The following chemicals were also used to obtain bis(cyclic carbonate): dimethylacetamide (DMAC), tetrabutylammonium bromide (TBAB) and acetone acquired from Sigma Aldrich (Germany, Poland). Compressed carbon dioxide (in a gas cylinder) was purchased from Messer (Kraków, Poland). 

### 3.2. Synthesis of BPA Bis(cyclic carbonate) under Conventional Conditions

An amount of 70 g of Epidian 6 and 5% mass TBAB were introduced into a three-necked flask equipped with a magnetic stirrer and a condenser. TBAB was used as a catalyst. The whole was heated to 105 °C and stirred to homogenize the mixture. Then, CO_2_ was introduced, the flow of which was recirculated using a peristaltic pump at a rate of 400 mL/min according to the rotameter readings. After 82 h, 10 mL of DMAC was added to the system due to its high viscosity. The reaction was carried out for 144 h and its progress was monitored using FT-IR spectroscopy. The obtained product was in the form of a white paste.

### 3.3. Synthesis of BPA Bis(cyclic carbonate) under Microwave Radiation Conditions

To the microwave reaction vial, 20 g of Epidian 6 and 5% mass TBAB were introduced. The whole mixture was placed in a Biotage^®^ (Uppsala, Sweden) Initiator+ microwave reactor and stirred by heating the system to 105 °C to obtain a homogeneous mixture. The reactor operates with a maximum power of 400 W from a magnetron at 2.45 GHz, and the appropriate amount of energy supplied was automatically controlled using the deflector (shutter) present in the reactor in order to maintain the set reaction temperature of 105 °C. CO_2_ (75 mL/min) was continuously introduced through a Teflon capillary. The reaction was carried out for 44 h and its progress was monitored using FT-IR spectroscopy. The product obtained was a white solid.

### 3.4. Purification of the Obtained Crude Bis(cyclic carbonates)

A sixfold excess of acetone (60 g) was added to raw BPA bis(cyclic carbonate) (10 g) to obtain a suspension, which was filtered to obtain 2 g of solid—fraction I. The same amount of water was added to the obtained filtrate (63 g) and fraction II was obtained in the amount of 6 g. Then, 100 g of water was added to the filtrate again and 1.5 g of precipitate was obtained—fraction III.

### 3.5. Methods

#### 3.5.1. FT-IR

Fourier-transform infrared spectroscopy (FTIR) was applied to track the reaction progress using a Thermo Scientific (Waltham, MA, USA) Nicolet iS5 spectrometer equipped with a diamond crystal in an attenuated total reflectance unit manufactured by Thermo Electron Corporation. FTIR analysis was conducted in the wavenumber range of 4000–400 cm^−1^, with a scanning resolution of 4 cm^−1^.

##### Estimation of CC mol Concentration in 100 g of Product

The estimation of cyclic carbonates was conducted by FTIR-ATR by surface measurement of the normalized 1786 cm^−1^ peak. The normalization was performed by dividing each product spectrum by the average of that spectrum; therefore, the amount of the measured substance and ATR contact did not play a crucial role in the peak intensity. The normalized peak area was compared to the calibration curve by adding different amounts of propylene carbonate to Epidian 6 (Table S1 in Supplementary Materials). The spectra of the obtained mixtures were recorded and normalized in the same manner as written above. Then, the 1786 cm^−1^ peak surface was compared with the known molar amount of CC in the mixture. From fitting, we found the empirical quadratic function from which we can calculate the molar amount of CC in 100 g of product (calibration curve measurement points are available in Figure S1 in Supplementary Materials):

$${CC}_{cont}=9.92\cdot 10^{-3}+5.07\cdot 10^{-4}\cdot {NA}_{1786}+4.76\cdot 10^{-7}\cdot {{NA}_{1786}}^{2}$$

where

*CC_cont_* is a molar amount of cyclic carbonate groups in 100 g of the product;
*NA*_1786_ is a normalized surface of the peak 1786 cm^−1^ from νC=O from cyclic carbonates.

#### 3.5.2. NMR

^13^C NMR spectra were recorded with an FT-NMR 500 MHz spectrometer (JEOL JNM-ECZR500 RS1, ECZR version (Peabody, MA, USA). The measurement temperature was 21 °C with a pulse width of 3.4 µs, 2 s of relaxation and 1.6 s of acquisition of 3072 scans for carbon ^13^C resonance. DMSO-d6 was used as a solvent and the spectra shift was referenced on their characteristic shift.

#### 3.5.3. TG

Thermal stability testing was performed using a TG 209 F1 Libra thermogravimetric analyzer from Netzsch (Houston, TX, USA). Samples weighing approximately 5 mg were placed in standard open crucibles made of aluminum oxide (α Al_2_O_3_). The measurement temperature range was 30–600 °C, with a heating rate of 10 °C/min. An inert nitrogen atmosphere was used for the measurements.

#### 3.5.4. GPC

Gel Permeation Chromatography (GPC) analyses were carried out in THF at 40 °C at a flow rate of 1 mL/min using a Shimadzu chromatograph (Duisburg, Germany) equipped with UV-Vis SPD-20 A/20 AV detector. A series of two Phenogel columns with a particle size of 5 μm and pore sizes of 50 Å was used. The elution times were converted to molecular weight by calibration with polystyrene (PS) standards.

## 4. Conclusions

Bis(cyclic carbonates) were successfully synthesized from a commercial epoxy resin based on bisphenol A and CO_2_ at atmospheric pressure under solvent-free conditions. Supporting the reaction with microwave irradiation resulted in a more than three-fold increase in the reaction rate compared to the reaction carried out using conventional heating, which can be explained by better heat distribution in the reaction mixture. Crude bis(cyclic carbonates) were isolated and purified to obtain three fractions differing in purity, which were analyzed using FTIR, NMR, TG and GPC methods. The tests showed that the best purity, i.e., the highest monomer content, was found in sample m1 obtained under microwave radiation. The FTIR results showed that samples obtained using the microwave method had a higher content of cyclic carbonates. However, the m1 fraction showed the highest CC content. Corresponding to m1, the c1 fraction obtained from the sample obtained under conventional conditions is characterized by lower purity due to the presence of a small amount of dimers and trimers. This may be due to the fact that in the final phase of the reaction from which fraction c1 was separated (conventional method), a small amount of solvent (DMAC) was added to reduce the viscosity of the reaction mixture. The presence of the solvent may probably cause problems with the separation of the highest purity fractions. The increased amount of oligomers and impurities had a negative impact on the thermal properties. The use of the microwave method for the synthesis of bis(cyclic carbonates) and the uniform temperature distribution thus obtained allowed for solvent-free synthesis, which had a direct impact on obtaining products of higher purity in a shorter time. The increased purity and homogeneity of the obtained fractions may have a positive effect on the phase separation of non-isocyanate polyurethanes (NIPUs) obtained from bis(cyclic carbonates).

## Figures and Tables

**Table 1:** Estimation of cyclocarbonate CC concentration in 100 g of products.

Sample	mol CC/100 g
m1	0.232
m2	0.218
m3	0.223
c1	0.161
c2	0.126
c3	0.132

**Table 2:** TG results of bis(cyclic carbonates) (in inert atmosphere).

Sample	T_onset_ (°C)	T_DTG_ _max1_ (°C)	T_DTG_ _max2_ (°C)	T_DTG_ _max3_ (°C)	DTG _max1_ (%/min)	DTG _max2_ (%/min)	DTG _max3_ (%/min)	m1 (%)	m2 (%)	m3 (%)	Residual Mass (%)
E6	276.5	-	310.0	396.6	-	−21.9	−2.3	-	−83.1	−15.8	1.0
m1	222.5	249.0	330.2	419.3	−2.3	−15.0	−3.6	−11.7	−63.8	−21.8	2.6
m2	219.1	246.6	330.4	405.4	−2.4	−15.1	−3.7	−10.6	−64.2	−23.4	1.8
m3	222.9	252.3	330.0	417.0	−2.2	−14.7	−4.2	−12.5	−63.8	−22.3	3.8
c1	211.7	240.6	338.1	426.2	−2.9	−10.0	−4.7	−15.6	−58.4	−23.6	2.4
c2	212.1	244.4	324.9	432.4	−2.1	−7.6	−7.2	−13.1	−43.0	−39.3	4.6
c3	215.4	246.9	318.1	415.0	−2.3	−6.3	−8.2	−10.1	−38.0	−42.7	6.8

T_onset_—extrapolated temperature of the beginning of thermal decomposition; T_DTGmax1_, T_DTGmax2_, T_DTGmax3_—temperatures of maximum rate of residual mass for the first and second stage; DTG_max1_, DTG_max2_, DTG_max3_—maximum rates of residual mass for the first and second stage; m1, m2, m3—residue mass for the first, second and third stage of thermal degradation.

**Table 3:** Analysis of molecular weights and determination of the shares of individual fractions using GPC.

Samples	**Peak I**	**Peak II**	**Peak III**
	M_n(theoretical)_ = 428 [g mol^−1^]	M_n(theoretical)_ = 712 [g mol^−1^]	M_n(theoretical)_ = 997 [g mol^−1^]
	M_n(experimental)_ = 504 [g mol^−1^]	M_n(experimental)_ = 731 [g mol^−1^]	M_n(experimental)_ = 1052 [g mol^−1^]
	**Mass fraction (%)**	**Mass fraction (%)**	**Mass fraction (%)**
m1	93.6	4.3	2.1
m2	78.5	15.4	6.1
m3	84.5	14.1	1.4

## Data Availability

Data are contained within the article and Supplementary Materials.

## Supplementary Materials

The following supporting information can be downloaded at https://www.mdpi.com/article/10.3390/molecules29010250/s1, Table S1. The calculated and measured parameters for the calibration. Figure S1. The calibration curve for the CC content estimation via FTIR method.
