# Supplementary material for: Synthesis of Bis(cyclic carbonates) from Epoxy Resin under Microwave Irradiation: The Structural Analysis and Evaluation of Thermal Properties
Source: Molecules. 2024 Jan 3;29(1):250. doi: 10.3390/molecules29010250 (PMC10781095; doi:10.3390/molecules29010250)
Supplement: Supplementary file 1 [file molecules-29-00250-s001.zip › molecules-2752520-supplementary.pdf]

Table S1. The calculated and measured parameters for the CC calibration.

m_{pCC} [g]	m_{E6} [g]	CC_{cont} mol pCC/100g	A_{1786} [%T]	AVG spectrum [%T]	NA_{1786} [%T]
0.029	1.996	0.014027806	0.364	0.0472	7.71
0.255	1.763	0.123775821	8.795	0.0471	186.60
0.478	1.552	0.230647449	15.723	0.0462	340.61
0.724	1.303	0.349865898	21.807	0.0471	463.48
0.916	1.017	0.464173578	25.054	0.0433	578.10
1.116	0.873	0.549599347	30.827	0.0470	655.87
1.366	0.636	0.668349184	35.444	0.0467	759.73
1.599	0.431	0.771559143	39.389	0.0469	840.21

m_{pCC} – mass content of propyl carbonate, **m_{E6}** – mass content of Epidian 6, **CC_{cont}** – the molar amount of cyclic carbonate groups per 100 g of the mixture, **A_{1786}** – the area of C=O peak (1786 cm^{-1}) for each measuring point, **AVG spectrum** – the average value of the full spectrum, **NA_{1786}** – normalized A_{1786} by dividing with average spectrum. Blue-colored columns were taken to the graph.

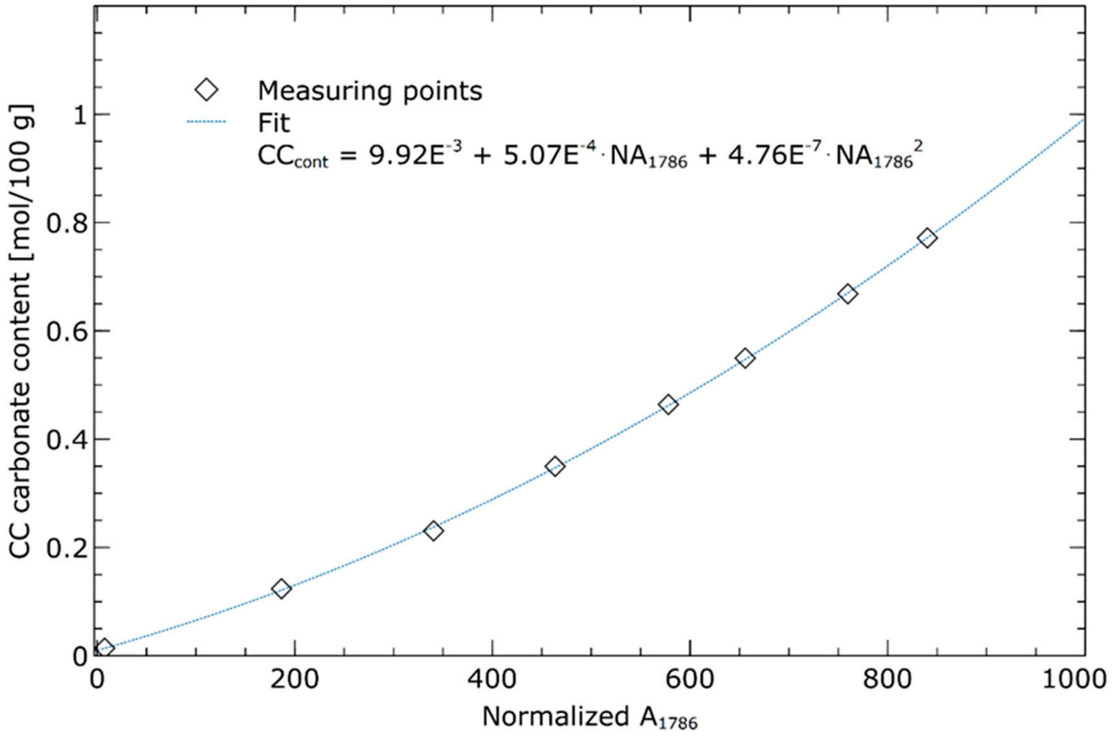

Figure S1. The calibration curve for the CC content estimation via FTIR method.
